# Survival on land: A dark-grown seedling searching for path

**DOI:** 10.3389/fpls.2023.1110521

**Published:** 2023-02-02

**Authors:** Hao Gong, Zhiwei Zeng, Léa Tessier, Leno Guzman, Zihao Yuan, Shuai Li, Wenhan Zheng, Ying Chen, Long Qi

**Affiliations:** ^1^ College of Engineering, South China Agricultural University, Guangzhou, Guangdong, China; ^2^ Department of Biosystems Engineering, University of Manitoba, Winnipeg, MB, Canada; ^3^ Department of Agricultural Engineering Technology, University of Wisconsin-River Falls, WI, River Falls, United States; ^4^ Department of Biological Sciences, University of Manitoba, Winnipeg, MB, Canada

**Keywords:** seedling, path, shape, growth medium, resistance, pore

## Abstract

To initiate its development into a plant, a small dark-grown seedling (prior to its emergence from the ground) must penetrate through the growth media. The path that the seedling takes during this journey has yet to be explained. As such, we conducted non-destructive tests using CT scans to observe the growth of dark-grown seedlings in soil over time; we also developed a model to simulate the dynamics of an emerging seedling, and to examine effects of various growth medium conditions, including Lunar soil. It was previously postulated that, with gravitropism in a terrestrial growth medium, a dark-grown seedling would grow directly upright. However, our CT scan results showed that dark-grown soybean seedlings departed from the vertical path in soil, as far as a lateral distance of approximately 10 mm. The phenomenon of the non-straight path was also demonstrated by the model results. Through simulations, we found that an emerging seedling naturally weaves through the particles of growth medium, in search for the path of least resistance. As a result, the seedling ends up travelling a longer distance. Compared with a seedling that was artificially forced to take a straight path in a growth media, the seedling taking the natural path encountered significantly lower resistances (20% lower) from the growth medium, while travelled 12% longer distance during the emergence process. A seedling encountered a much higher impedance in Lunar soil. Our results suggest that taking the path of least resistance, in addition to shaping and orientating itself for mechanical advantage, are strategies evolved by plant species that have contributed to its vast success. An understanding of plant behavior and survival strategies on Earth lay the foundation for future research in agriculture in novel environments, including on celestial bodies.

## 1 Introduction

The process from a seed to a plant consists of multiple phases, including the emergence phase. Emergence involves seedling growth inside the body of growth medium prior to any photosynthesis. Thus, the seedling during the emergence phase is referred as the “dark-grown seedling” (Gupta et al., 2012). In addition to appropriate environmental conditions (e.g. water and temperature), the seedling must be able to push through the growing medium, which varies in hardness and porosity in nature. Seed plants have been extremely successful in propagating on the earth, with an estimated 223,300 to 421,986 species to date (Scotland and Wortley, 2003). Given their widespread success in survival, these dark-grown seedlings must have evolved a means to search for a path leading to the plant’s emergence. It thus begs the questions: how does a dark-grown seedling select its path through the growth medium?

Answering this question is critical for understanding the adaptive strategies of plants in variable terrestrial soils. These elucidations would also have important implications for understanding whether extra-terrestrial soils are habitable for plants, considering the fact that plants require the same basic needs for growth on celestial soil as they do on Earth (Oluwafemi et al., 2018). In order to maintain sustenance in space, habitants must grow their own crops (Oluwafemi et al., 2018). Although soilless options, including hydroponic and aeroponic growing systems, have been suggested for space farming, neither systems have the capability of absorbing the inedible part of plants and wastes from habitants and food processing. As such, there is an increasing interest toward “soil-based” space agriculture, making use of regolith, lunar, or Martian soil (Yamashita et al., 2009). Such a system is more sustainable due to its potential to compost organic residues from roots and plant residue, decompose organic waste, and filter water (Maggi and Pallud, 2010). An understanding of plant survival strategies in relation to the growth medium in terrestrial soils will serve as the foundation for future research on plant survival in extra-terrestrial soils.

In the terrestrial environment, a dark-grown seedling would grow directly upright within the growth medium, based on gravity-dictated directional growth (Hangarter, 1997). However, in reality, seedlings deviate from their straight path. In studying the effects of soil surface crust on the emergence of soybean seedlings, Rathore et al. (1981) speculated that the departed soybean seedlings may have followed the path of least resistance. To date, this statement is still at the hypothesis stage and has never been proven, largely due to experimental obstacles that would prove challenging. The main challenge is that the growth of a dark-grown seedling occurs inside the body of growth medium. Physical observations of the seedling growth require to open the medium material, which would disturb to the natural system. Fortunately, methods of non-destructive tests such as computed tomography (CT) scanning are available and has been used to observe plant root systems (Lontoc-Roy et al., 2006; Han et al., 2008). Using this method, the emergence process of a dark-grown seedling can be observed without disturbing the medium material.

Another challenge was that the dynamics of an emerging seedling occurs in a relatively small zone, i.e. around a seedling in a non-homogeneous growth medium. Measurements of such a complex system in a micro-scale would be difficult, if not possible. Fortunately, the Discrete Element Method (DEM) (Cundall and Strack, 1979), a numerical modelling and simulation tool, is available to simulate complex systems in micro scales, such as soil particle-particle and soil-seedling interactions in the process of seedling emergence. In DEM, growth medium can be modelled as an assembly of discrete particles. If an external force exerts on the assembly, some individual particles will displace, causing contact forces between the particles. The force-displacement regime is governed by some constitutive laws, also named as contact laws, such as the Newton second law. Behavior of a DEM model is dependent on the contact laws used and the model input parameters. Examples of DEM model parameters include particle stiffness and friction. In modelling using DEM, the contact law and model parameters should be carefully selected, so that the model behavior represents the reality. The best way to determine the model parameters is calibration.

DEM has been widely used to simulate soil dynamics in agriculture, such as soil-machine interaction in the areas of tillage and seeding. Lately, few DEM models were developed to simulate soil-seedling interaction in the process of seedling emergence. These models aimed to predict the soil resistances to soybean seedlings (Gong et al., 2019; Zeng et al., 2020), and to canola seedlings under different soil compaction levels (Gong et al., 2022). However, these existing simulations had the assumption that seedlings grow in a fixed straight vertical path within the soil, which is not realistic.

Here we aimed to fill the aforementioned existing gaps using the soybean as a case study. The objectives were to (1) use non-destructive tests (CT scans) to examine the growth direction and the morphological changes of dark-grown seedlings during the emergence process, (2) simulate the natural path of a dark-grown seedling using the DEM, and (3) examine how a dark-grown seedling survived under various growth medium conditions, including extra-terrestrial soil. The results enable us to make connections between behaviors of plants and their adaptive strategies.

## 2 Material and methods

### 2.1 Experiments

#### 2.1.1 CT scanning of soybean emergence process and image analyses

The emergence process of soybean (*Glycine max* (L.)) seedlings in soil was observed using non-destructive tests (3D CT scanning). The soybean seeds (variety: Thunder) and soil (sandy loam: 70% sand, 16% silt, and 14% clay) were from Manitoba, Canada. This soil was selected because its texture is between a clayed soil and a sandy soil which are the two extreme soil textures. Containers (a base diameter of 46.1 mm, a top diameter of 60.5 mm, and a height of 63.8 mm) were filled with the soil. Each container was seeded with one soybean seed at a target depth of 30 mm (Cox and Cherney, 2016). In total, 10 containers were seeded and were labelled Test No. 1 to Test No. 10. The soil in the 10 containers had an average soil moisture content of 24.7% (dry basis) and an average soil bulk density of 1433 kg m^-3^ measured according to ASABE Standards (2015). After seeding, the seeded containers were scanned immediately and at 12 hr intervals using a 3D CT scanner (Bruker SkyScan 1275; specifications:<5mm px size, 3Mpx FP camera, and 20-100 kV source). Between scanning intervals, the containers were put in an environmental chamber that had a controlled temperature of 28°C (Tyagi and Tripathi, 1983) and a relative humidity of 75%. Scanning continued until the seedlings emerged from the soil.

**Figure 1:**
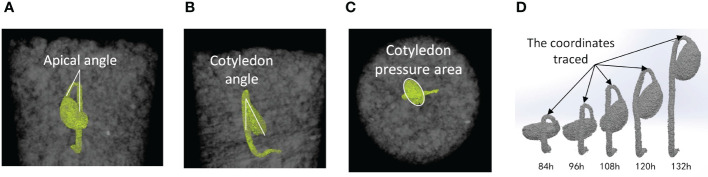
Examples of CT images of soybean seedling at time (t); images from Test No. 5. **(A)** Apical angle of hypocotyl in the X-Z plane at t=108 (h) **(B)** Rotation angle of cotyledon in the Y-Z plane at t=108 (h) **(C)** Pressure area of cotyledon in the X-Y plane at t=108 (h) **(D)** Locations on hypocotyl where the coordinates were monitored to trace the direction of seedling growth.

The CT images were analyzed using an imaging analysis software (Avizo, Themo Fisher Scientific, Waltham, Massachusetts, USA) to extract morphological characteristics of seedlings from the time of hypocotyl formation up to the completion of the emergence phase. The apical angle of the hypocotyl was determined on the X-Z plane of image (**Figure 1A**). The rotation angle of the cotyledon was obtained as the angle formed by the longest axis of the cotyledon and the vertical direction in the Y-Z plane (**Figure 1B**). Pressure area of the cotyledon was the area of the cotyledon projected vertically to the horizontal plane (X-Y plane) (**Figure 1C**). Also, the growth direction was quantified by tracing the coordinates of the apex of the hypocotyl (**Figure 1D**).

#### 2.1.2 Seedling emergence experiments under different growth medium conditions

**Figure 2:**
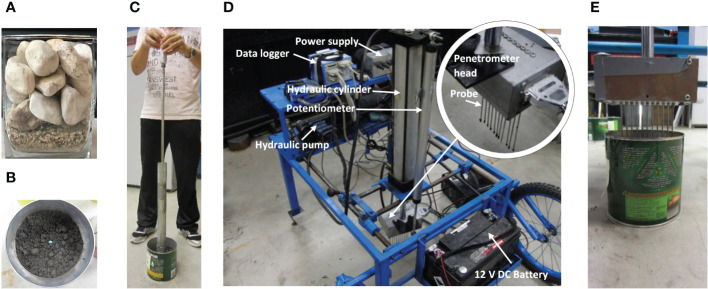
Experiments. **(A)** Emergence test with gravel as the growth medium. **(B)** Emergence test with soil as the growth medium, showing the soybean seed planted. **(C)** Soil being compacted by a proctor. **(D)** Micro-penetrometer. **(E)** Probes penetrating the soil for measurements of soil cone indices.

To examine growth medium effects on seedling emergence, two types of growth medium, compacted soil and gravel, were used. They represented two scenarios of pore availability and penetrability. The experiment of using gravel pieces (equivalent diameter range: 15-30 mm) as the growth medium was performed with six replicates. First, a layer (12 mm) of vermiculite was placed at the bottom of container (65x65 mm in cross-section and 82 mm in height) as a seedbed (**Figure 2A**). A small amount of water was added to the vermiculite. A soybean seed was then pressed into the seedbed. This helped the seed germination and root development. Then, the container was filled with a 60-mm layer of gravel pieces. The gravel in the containers was not compacted. The seeded containers were covered with a thick blanket, and the seedling emergence situation was observed.

The experiments using compacted soil as the growth medium had a completely randomized design with three treatments: Low, Medium, and High soil compaction levels. Each treatment was replicated three times, giving a total of 9 tests (3 compaction levels x 3 replicates). The experiment followed the following procedures. First, half the container (157 mm in height and 152 mm in diameter) was filled with soil. Then, a soybean seed was placed on the soil surface (**Figure 2B**). The seed was covered with a predetermined amount of soil to ensure a final seeding depth of 30 mm. Finally, the soil was compacted by dropping the hammer of a soil proctor (with a 4.5 kg dropping hammer and a free fall of 450 mm) (**Figure 2C**). For the Low, Medium, and High soil compaction treatments, one, two, and three hammer drops were applied, respectively. After compaction, the average bulk densities of the three compaction levels were: 1284, 1341, and 1455 kg m^-3^, respectively. The seeded containers were covered with a wet cloth to reduce moisture evaporation from the soil. The emergence of the plant was then allowed to take place.

The aforementioned soil compaction levels were quantified in terms of soil cone index, which is measured by vertically pushing a cone penetrometer into the soil while recording the pressure on the cone (named as cone index) in the vertical direction (Morton and Buchele, 1960; Dexter and Hewitt, 1978). For this, a total of 9 dummy containers (3 compaction levels x 3 replicates) were prepared. The soil in the dummy containers was compacted in the same method as the seeded containers, except that the dummy containers were not seeded. Soil cone indices in dummy containers were measured using a micro-penetrometer (**Figure 2D**). The penetrometer head had 11 probes spaced 10 mm apart. Each probe was a 165-mm long rod with a cone at the end. The cone had an angle of 57° and a base diameter of 2.75 mm. A light sleeve tube (2.75 mm in diameter) was set outside of the rod to minimize friction between the rod and the soil. The other end of the probe was connected to a load cell (capacity: 22 N, Thin Beam Load Cell, Model: LCL-005, Omega Environmental, QC, Canada). During the measurement, the probes penetrate the soil in container to a depth of 30 mm at a speed of 3x10^-4^ m s^-1^ (**Figure 2E**), and the cone index signals of the load cells were recorded by a data logger (InstruNet, Omega Environmental, CT, USA) at 5 Hz. At the same time, a potentiometer measured the distance (mm) that the probe travelled. Eleven probes gave 11 index-depth curves for each penetration measurement.

### 2.2 Modelling and simulation

#### 2.2.1 Development of a seedling emergence model

**Figure 3:**
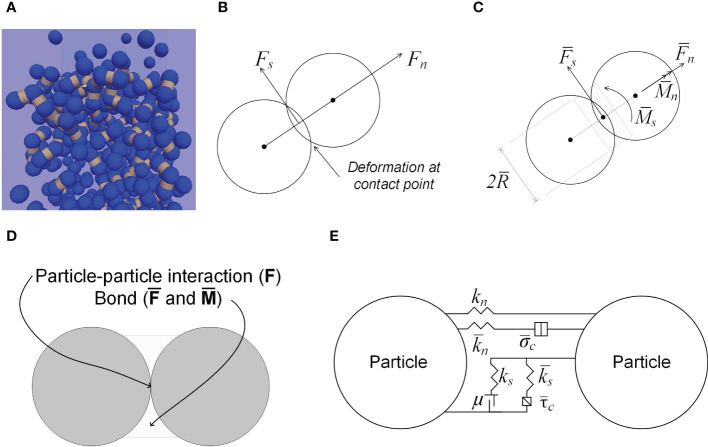
Modelling of growth medium. **(A)** Growth medium particles (blue) and bonds (dark yellow bands) between particles in contact. **(B)** Contact forces between particles. **(C)** Bond forces and bond radius. **(D)** Forces resulting from the particle-particle interaction. **(E)** Linear Parallel Contact Bond model.


A seedling emergence model was developed using DEM software: Particle Flow Codes in Three Dimension (PFC^3D^) (Itasca Consulting Group, Inc. Minneapolis, USA). The model simulated the growth of a soybean seedling in a growth medium. The growth medium was modelled as an assembly of spherical particles, and bonds were added between particles in contact (**Figure 3A**) to mimic the cohesion between particles in a natural growth medium. The particle sizes and the DEM parameters could be varied to represent various growth media. The contacts between particles followed the linear parallel bond contact law implemented in PFC^3D^ (Potyondy and Cundall, 2004). In this contact law, force (F) arises at the particle-particle contact in both normal and shear directions (**Figure 3B**). Bond between two particles is able to withstand force ( $\bar{F}$ ) and moment ( $\bar{M}$ ) in both normal and shear directions (**Figure 3C**). Using this contact law, the force regime at the interface between two particles includes F, $\bar{F}$ , and $\bar{M}$ , as illustrated in **Figure 3D**. The particle-particle contact forces are the results of particle deformations, as described in the following equations (Potyondy and Cundall, 2004):

(1) $$F_{n}{=k}_{n}U_{n}$$

(2) $$\Delta F_{s}=k_{s}\Delta U_{s}$$

where *F_n_* and *F_s_* are the particle-particle contact forces in the normal and shear directions respectively (**Figure 3B**); the corresponding deformations are *U_n_* and *U_s_*, and the corresponding particle stiffness are *k_n_* and *k_s_*. The bond forces are the results of translational and angular deformation of the bond, as described in the following equations (Potyondy and Cundall, 2004):


(3) $$\Delta {\bar{F}}_{n}={\bar{k}}_{n}A\Delta U_{n}$$

(4) $$\Delta {\bar{F}}_{s}=-{\bar{k}}_{s}A\Delta U_{s}$$

(5) $$\Delta {\bar{M}}_{n}=-{\bar{k}}_{s}J\Delta \theta_{n}$$

(6) $$\Delta {\bar{M}}_{s}=-{\bar{k}}_{n}I\Delta \theta_{s}$$

where $\bar{F_{n}}$ and $\bar{F_{s}}$ are bond forces in the normal and shear directions (**Figure 3C**); the corresponding moments are $\bar{M_{n}}$ and $\bar{M_{s}}$ (**Figure 3C**), and the corresponding bond stiffness are $\bar{k_{n}}$ and $\bar{k_{s}}$ . *A* is cross-sectional area of the bond; *I* is area moment of inertia; *J* is polar moment of inertia; *θ_n_* and *θ_s_* are angular deformations.

At the contact, particles will slip, when:


(7) $${\text{F}}_{\text{s}}\leq {\text{ µF}}_{\text{n}}$$

where *μ* is particle friction coefficient. Bond will break if the maximum stress of the bond (caused by external force) is greater than the bond strength specified. These statements are equivalent to the following equations (Potyondy and Cundall, 2004):


(8) $${\bar{\sigma }}_{max}=\frac{-{\bar{F}}_{n}}{A}+\frac{\left|{\bar{M}}_{s}\right|\bar{R}}{I}<{\bar{\sigma }}_{c}$$

(9) $${\bar{\tau }}_{max}=\frac{\left|{\bar{F}}_{s}\right|}{A}+\frac{\left|{\bar{M}}_{n}\right|\bar{R}}{J}<{\bar{\tau }}_{c}$$

where $\bar{R}$ is bond radius (**Figure 3C**); ${\bar{\sigma }}_{max}$ and ${\bar{\tau }}_{max}$ are the normal and shear stresses in the bond respectively. ${\bar{\sigma }}_{c}$ and ${\bar{\tau }}_{c}$ are the normal and shear strength of the bond respectively.

In simulations, the aforementioned parameters of particle and bond need to be input in the model. The roles of the parameters are illustrated in **Figure 3E**. Particle or bond stiffness functions as a spring that controls the deformation in both the normal and shear directions; the friction coefficient function as a slider controlling sliding between two particles in shear direction; bond provides cohesion between particles; additional parameters (*β_n_* and *β_s_*), viscous damping in the normal and shear directions, function as a dashpot to ensures that the system is stable in DEM simulations which are dynamic in nature.

When using soil as the growth medium, the DEM parameters (**Table 1**) were adopted from a previous study that used the same soil (Sadek and Chen, 2015). A local damping coefficient (0.5) was introduced to further calm down the particle dynamics. The bond radius ( $\bar{R}$ ) was set to be 50% of the particle radius. Preliminary simulations showed that model outputs were sensitive to the medium particle normal stiffness (*k_n_*). Thus, this parameter was calibrated as described later in the paper.

**Table 1:** Input parameters for model soil and model cotyledon.

Model parameter	Symbol	Unit	Value
**Soil particle normal stiffness**	*k_n_*	N m^-1^	To be calibrated
**Soil particle shear stiffness**	*k_s_*	N m^-1^	*k_s_ = k_n_*
**Soil particle internal friction coeff.**	*µ*	–	0.5
**Soil viscous normal damping coeff.**	*β_n_*	–	1.0
**Soil viscous shear damping coeff.**	*β_s_*	–	1.0
**Soil bond normal stiffness**	${\bar{k}}_{n}$	Pa m^-1^	3.57x10^10^
**Soil bond shear stiffness**	${\bar{k}}_{s}$	Pa m^-1^	3.57x10^10^
**Soil bond tensile-strength**	${\bar{\sigma }}_{c}$	Pa	2x10^4^
**Soil bond shear-strength**	${\bar{\tau }}_{c}$	Pa	2x10^4^
**Seedling particle density**	*ρ_seed_*	Kg m^-3^	1228
**Seedling modulus of elasticity**	*E_seed_*	Pa	1.04x10^8^
**Seedling internal friction coeff.**	*µ_seed_*	–	0.45
**Seedling normal stiffness**	*k_n-seed_*	N m^-1^	1.87x10^6^
**Seedling shear stiffness**	*k_s-seed_*	N m^-1^	1.87x10^6^

**Figure 4:**
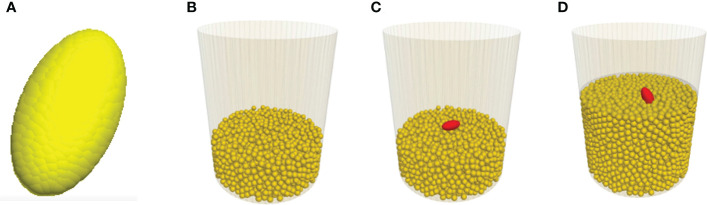
Screenshots showing various stages of the modelling. **(A)** Model cotyledon. **(B)** Medium particles as the seedbed. **(C)** Initial position of cotyledon before being covered with medium particles. **(D)** After being covered, cotyledon emerged from the medium.

The soybean seedling was simplified to be a cotyledon, since the large pressure area at the cotyledon would dictate the resistance that the seedling experiences in a growth medium. Based on the CT images, the cotyledon has a shape close to an ellipsoid. Thus, the model cotyledon was simplified as an ellipsoid and it was constructed as a clump of spherical particles (**Figure 4A**). The required DEM parameters (**Table 1**) for the model cotyledon were adopted from Boac et al. (2009). The normal stiffness (*k_n-seed_*) was derived from *E_seed_* (Boac et al., 2009) using the relationship: *k_n-seed_=4RE_seed_* (Itasca, 2022). The other DEM parameter, shear stiffness (*k_s-seed_*) was assumed to be equal to *k_n-seed_* (Zhou et al., 2014).

To model the emergence process of a seedling in a medium, a container with the same size as those used in the CT scan tests was created. A layer of medium particles was generated in the container to represent the seedbed (**Figure 4B**). Then, the model cotyledon was placed on top of the seedbed in the middle of the container (**Figure 4C**). Finally, the cotyledon was covered by a layer of medium particles. If desired, the medium particles could be compressed by moving a horizontal plate downward from the medium surface to achieve a specific bulk density of the medium. Next, to simulate the growth of the seedling, the cotyledon was given a motion. The simulation stopped when the cotyledon had travelled to the surface of the growth medium (**Figure 4D**).

#### 2.2.2 Calibration of the seedling emergence model

**Figure 5:**
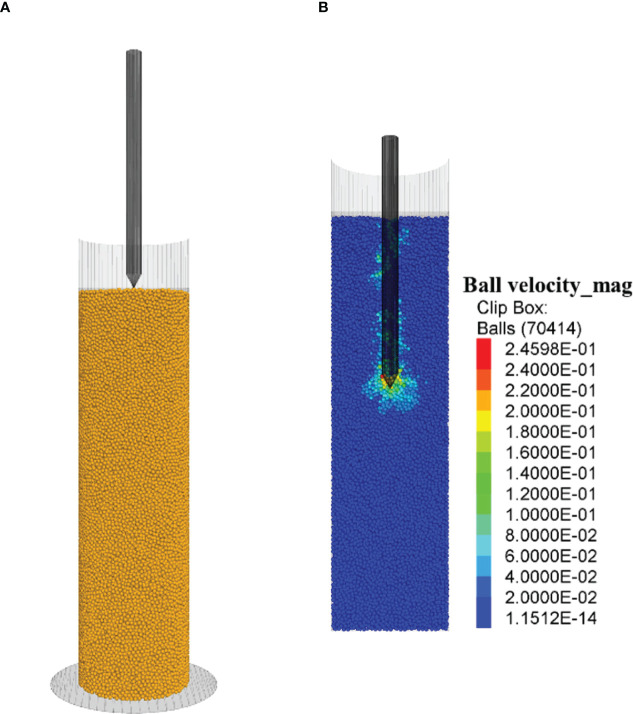
Virtual soil penetration tests. **(A)** Initial position of the micro-penetrometer probe and soil particles. **(B)** Soil particle velocity countours, while the probe penetrating the soil.

Calibration of *k_n_*, the most influential DEM parameter, was done by comparing soil cone indices between virtual soil penetration tests and the aforementioned micro-penetrometer tests under the Low compaction soil. The virtual soil penetration tests were performed using PFC^3D^. In the virtual tests, the micro-penetrometer was simplified as a single probe formed by a cone and a rod (**Figure 5A**). A total of 70,414 soil particles (0.7 mm in diameter) was generated in a container (a diameter of 20 mm and a height of 80 mm). The bulk density of the model soil was matched with that of the real soil in the soil penetration tests, through adjusting the particle density of the model soil. The probe was initially positioned at the surface (**Figure 5A**). Then, the probe was given a downward speed to penetrate the soil to a depth of 30 mm (**Figure 5B**). All the conditions were the same as the real soil penetration tests, except for the penetration speed (0.3 m s^-1^), which was higher. Lower speeds require unpractically long computation time (Zeng and Chen, 2016).

While the probe moved down in the soil, the contact pressure in the vertical direction between the cone and soil particles was monitored as the predicted value of soil cone index. For the model calibration, the virtual test was run using the soil parameters listed in **Table 1** and an assumed value for *k_n_* to predict soil cone indices. The virtual test was repeated by adjusting *k_n_* until the predicted soil cone indices matched with the measured ones from the real soil penetration tests.

#### 2.2.3 Simulations of the natural path of a dark-grown seedling

Using the calibrated seedling emergence model, the natural path of a dark-grown seedling was simulated. The cotyledon was assigned a vertical speed in the Z direction only, which allowed the cotyledon to freely move naturally in the lateral directions. Simultaneously, the cotyledon was made to rotate about the shortest axis of the cotyledon (the Y-axis in this case), as observed in CT scans. To serve as a comparison, a straight path scenario was simulated, where the cotyledon was artificially forced to move upward vertically. This was achieved by assigning a vertical speed to the cotyledon in the Z direction and zero speeds in X and Y directions. The simulations for both scenarios were performed under the same medium particle diameter (2 mm) and other conditions.

As the cotyledon moved, the seedling trajectory in the medium was traced and contact forces between the cotyledon and soil particles were recorded. The sum of the contact forces in a direction is the resistance to the seedling from the medium in that direction. The total resistance was calculated from the resistance force components in the X, Y, and Z directions. The cotyledon motion resulted in the changes of strain energy stored at the particle contacts, and the changes in the system kinetic energy. Both strain energy and kinetic energy were tracked using the energy tracking features implemented in PFC^3D^ (Itasca, 2022).

#### 2.2.4 Simulations of the effects of various growth media

The calibrated seedling emergence model was also used to examine effects of various growth media. Various growth media were created using different particle sizes (particle diameter: 2, 4, 6, 8, and 10 mm), providing different porosities and pores of various sizes. This allowed for examination of the dynamic behaviors of seedling in response to these different conditions. In the simulations, the trajectories and resistances of the cotyledon was monitored in the method mentioned above.

#### 2.2.5 Simulation of extra-terrestrial soil-seedling interaction

The emergence model was applied to extra-terrestrial soils. As more information is available for Lunar soil than Martian soil, a simulation was run for Lunar soil. Lunar soil contains mainly medium sands and fine silt (Carrier, 2005). Compared with territorial soil particles, Lunar soil particles are more irregular. The size of basic particles varied from 0.020 to 0.500 mm. On the surface layer of the Moon, the average bulk density is 1500 kg/m^3^, the particle density is 3100 kg/m^3^, and the gravity is 1.62 m/s^2^. Despite the highly irregular shape of Lunar soil particles, it can still be assumed as assemblies of spherical particles in DEM simulations (Khademian et al., 2019). The particle size was set to be the upper value of the Lunar soil, 0.500 mm due to the limitations of the DEM method and computation power. In simulating the mechanical properties of Lunar soil using the DEM, Khademian et al. (2019) also used a larger size range. Although there is no water or organic matter in Lunar soil, its cohesion is high due to the interlocking among the angular particles. Therefore, Lunar soil has high shear strength, and thus the parallel bond model was used (Khademian et al., 2019). PFC model parameters were adopted from Khademian et al. (2019), and they were 3.2x10^12^ Pa for particle and bond effective modulus, 1.0 for normal-to-shear stiffness ratio, 2.2x10^9^ Pa for bond tensile strength and cohesion, 0.42 for particle friction coefficient, and 0.28 for normal and shear viscous damping ratio.

Preliminary simulations showed that running the model took an unpractically long time, if the low emergence speed of 0.015 m/s for terrestrial soil was used. In order to complete the simulation of Lunar soil in a practical time frame, the emergence speed was increased to 15 m/s. It was expected that such a huge increase would significantly affect the model outputs of soil impedance and path trajectory. To correct the speed effects, two simulations were run at larger particle diameter (2 mm), with one simulation using 0.015 m/s emergence speed and the other simulation using 15 m/s. Results showed that the soil impedance of the higher speed was 60.8 times that of the lower speed, and the path trajectory length of the higher speed was 92.4% of that at the lower speed. These two values were used as the correction factors. The reported simulation results from the Lunar soil have been corrected using these correction factors.

### 2.3 Statistical analysis

Statistical analyses were performed on simulation results in addition to measurement data. For each simulation run, particle distributions in the growth medium was randomized using the feature of randomization in PFC^3D^. Thus, a simulation of each condition could be replicated with randomly distributed medium particles, to mimic real life. Four replicates were performed for each simulation condition. With results from different replicates, statistical analysis could be performed on the simulation results. ANOVA was used for analyses on simulation results and the measurement data to examine the effects of the affecting factors using SAS (2021). Simulation results included growth medium resistance to seedling and trajectory length of seedling, under the natural path and straight path, and different soil porosities. Measurement data included soil cone indices obtained from the soil penetration tests under different soil compaction levels. Differences between treatments or levels were compared using Duncan Multiple Range tests at a significant level of P<0.05.

## 3 Results

### 3.1 Observations from CT scans

#### 3.1.1 Taking shape

**Figure 6:**
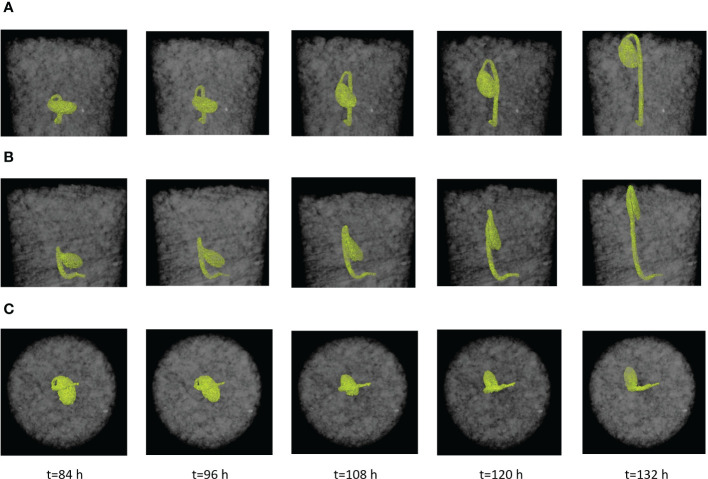
Time (t) lapse of a dark-grown soybean seedling in soil from CT images and morphological the characteristics of seedlings over time (Test No. 5). **(A)** X-Z view, the definition of apical angle of hypocotyl. **(B)** Y-Z view, the definition of cotyledon angle. **(C)** X-Y, the definition of cotyledon pressure area.

The transformation of the soybean seed to a seedling within soil was represented by the formation of a hypocotyl apex around t=84 h, followed by elongation of the hypocotyl. The hypocotyl pulls the cotyledon upward to the soil surface. At 132 h, 8 out of 10 seedlings emerged from the surface, as shown by the images in the X-Z plane (**Figure 6A**) and Y-Z plane (**Figure 6B**). Once the cotyledon arrives at the soil surface, it marks the completion of the dark-growth stage. The cotyledon rotated as it moved up in soil. Its orientation shifted from its initial horizontal position (the position at planting) to a nearly vertical position when arriving at the soil surface (the position at emergence) (**Figures 6A, B**). This action resulted in a reduced pressure area (projected area of cotyledon to the vertical direction) over time, as shown in the images of X-Y plane (**Figure 6C**).

**Figure 7:**
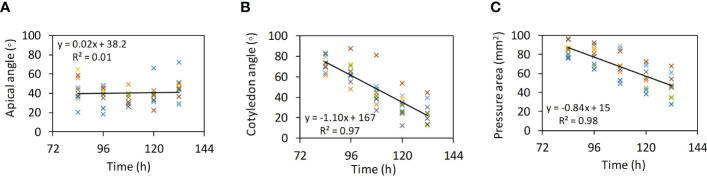
Morphological the characteristics of seedlings over time (Test Nos. 1 to 10). **(A)** Apical angles. **(B)** Cotyledon angles. **(C)** Pressure area.

The measured apical angle for 10 seedlings scattered around a constant trend over time (mean: 40.5°; SD: 11.4°) (**Figure 7A**). A smaller angle would provide a more pointed forefront that facilitates the penetration of the hypocotyl. The acuity of this angle may be limited as to avoid self-injury from excessive bending of the hypocotyl. The cotyledon angle decreased as the seedlings were approaching the soil surface in a linear trend (**Figure 7B**). Similarly, the pressure area also decreased (**Figure 7C**).

#### 3.1.2 Taking path

**Figure 8:**
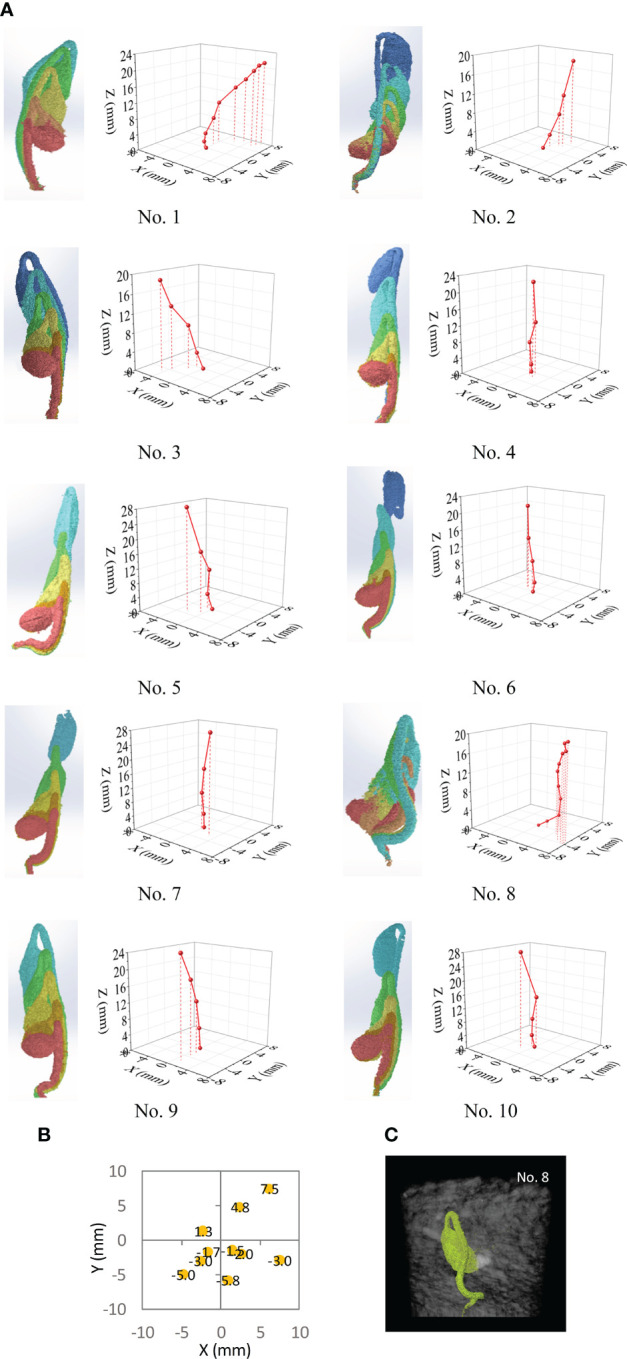
Analysis results from CT images and the path characteristics of 10 seedling over time. **(A)** Overlapped images of seedling and measured locations of hypocotyl apex of the seedling at different times. **(B)** Final location of hypocotyl apex on the X-Y plane at the time of emergence for 10 seedlings; the initial position was at the origin of X-Y plane. **(C)** A scan image of seedling from Test No. 8.

Although all soybean seedlings have a general upward growth trend due to the gravitropic response, their growth directions were not completely vertical. This is illustrated by the overlapping seedling images, which were in-situ, as well as by the measured position of the hypocotyl apex at each scanning time (**Figure 8A**). The change in apex position showed that the seedlings significantly departed from the vertical line. None of the paths among the 10 seedlings were the same. The random paths may be attributed to the non-homogeneous nature of the soil structure. Among the 10 seedlings, the lateral departure distances (the final locations relative to the initial location: X=0 mm and Y=0 mm) varied from 2.12 to 9.66 mm (**Figure 8B**). One extreme case was seedling No. 8 (**Figure 8C**), where bending occurred several times. Bending occurs within large voids in the soil (Arndt, 1965) or when large obstacles are encountered, similar to the concept of root buckling (Bizet et al., 2016). This case had more curved paths, possibly owing to more complex pore distribution in the soil. As the result, seedling No. 8 took 192 h to emerge, which was notably longer than the 132 h taken by the other seedlings.

### 3.2 Model simulation results

#### 3.2.1 Simulation conditions and calibrated soil stiffness

In the simulations, the dimensions of the model cotyledon were set as14.7, 8.2, and 6.0 mm for the length, width, and thickness, respectively, based on the averages of the 10 tests in the CT scans at t=108 h (the middle stage of seedling growth). The motion of the cotyledon in the soil needed to be set for the simulations. The CT scans showed that majority of the cotyledon took 48 h to reach the surface, and the cotyledon had rotated approximately 90°. With these observations, the estimated upward velocity of cotyledon was 1.97x10^-7^ m s^-1^, and the estimated rational speed of cotyledon was 9.09x10^-6^ rad s^-1^. Using these real-life speeds in the simulations would take unpractically long computing times. Thus, a higher upward velocity (0.015 m s^-1^) was used in the simulations (Zeng et al., 2020), and accordingly, the rotational speed was determined to be 0.785 rad s^-1^.

**Figure 9:**
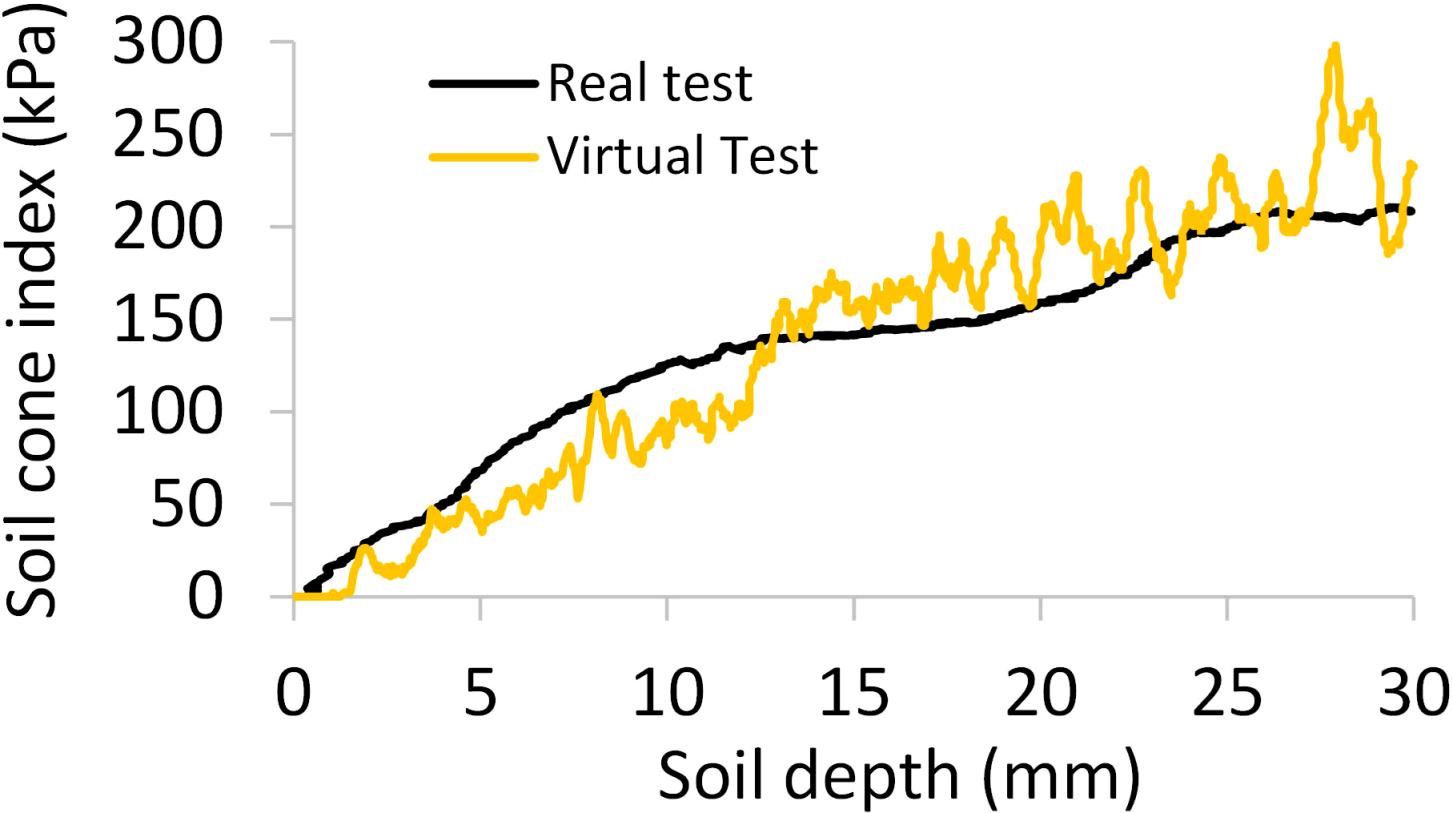
Comparisons of the simulated and measured curves of soil cone index-depth; averaged over three replicates.

The calibration results showed that the soil stiffness (*k_n_*) value of 4.0x10^3^ N m^-1^ produced the best match in soil cone indices between the real and virtual soil penetration tests. With this *k_n_* value, the cone index-depth curves from the virtual tests had similar trends as those from the real tests (**Figure 9**). The agreement between the virtual and real tests was further assessed by comparing the average cone indices (averaged over the depth of 0-30 mm), and the relative error was 2.0% only.

#### 3.2.2 The search for a path

**Figure 10:**
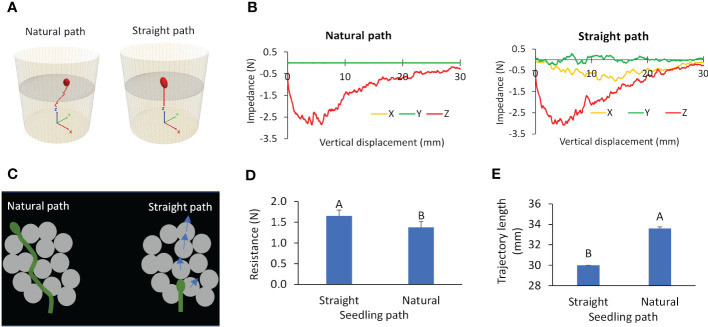
Simulated paths and resistances for two scenarios: natural path and straight path. **(A)** Trajectory. **(B)** Resistances to cotyledon. **(C)** Illustration of a growing seedling in growth medium. **(D, E)** Average total resistance and length of trajectory respectively; values labeled with different letters are statistically different according to Duncan’s Multiple Range Test at P<0.05.

Through simulations using the calibrated seedling emergence model, we found that the natural path of a dark-grown seedling (represented by the cotyledon) was a waved trajectory, in contrast to the forced straight path (**Figure 10A**). This finding was consistent with the natural path observed in the CT scan study. For the natural path, the resistance that the seedling encountered was primarily in the Z direction, and those in the X and Y directions were near zero (**Figure 10B**). Whereas, for the straight path, the cotyledon experienced higher resistance forces in all the directions (X, Y, Z). These results are explained using **Figure 10C**. In the natural path, the cotyledon moved through pores in the growth medium, taking the path of least resistance. In the straight path scenario, the cotyledon is forced to push through the overlaying growth medium particles along the way. As the results, the cotyledon encountered a significantly higher (by 20%) total resistance (**Figure 10D**). Also, when compared to the straight path, the cotyledon in the natural path travelled a significantly longer distance (by 12%) (**Figure 10E**). These results suggest that a dark-grown seedling departs from the vertical direction in order to find a path of least resistance, and that path is not necessarily the shortest.

**Figure 11:**
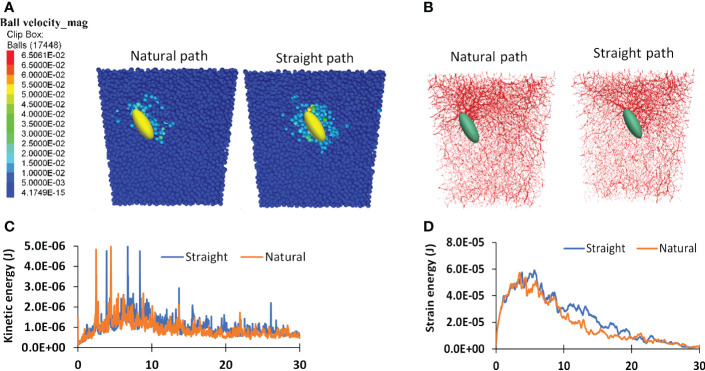
Simulated dynamic attributes for two scenarios: natural path and straight path. **(A)** Particle velocity contours of medium particles. **(B)** contact force chains within the medium particles. **(C, D)** Kinetic and strain energies of the system respectively.

Differences in the growth medium resistance between the natural path and the straight path can be further understood by examining the dynamics of cotyledon-medium interaction. A cotyledon taking the straight path pushed more medium particles as shown by the velocity contours of medium particles (**Figure 11A**). It also caused more medium particles being in contact, as shown by the soil contact force chains near the cotyledon (**Figure 11B**). As the results, medium particles impacted by the straight growing seedling have higher velocities, and therefore have higher kinetic energy in the system (**Figure 11C**). Higher contact forces between medium particles implies more strain energy was stored within the medium particles. This explains the higher strain energy in the straight path scenario (**Figure 11D**). Over a 30-mm growth medium depth, the average kinetic and strain energies of medium particles for the straight path were 10.8x10^-7^ and 4.83x10^-5^ J respectively, and the corresponding values for the natural path were 13.3% and 9.91% lower. The less dynamic responses of the soil caused by the seedling growth in the natural path explains its lower resistance.

#### 3.2.3 Effects of growth medium conditions

**Figure 12:**
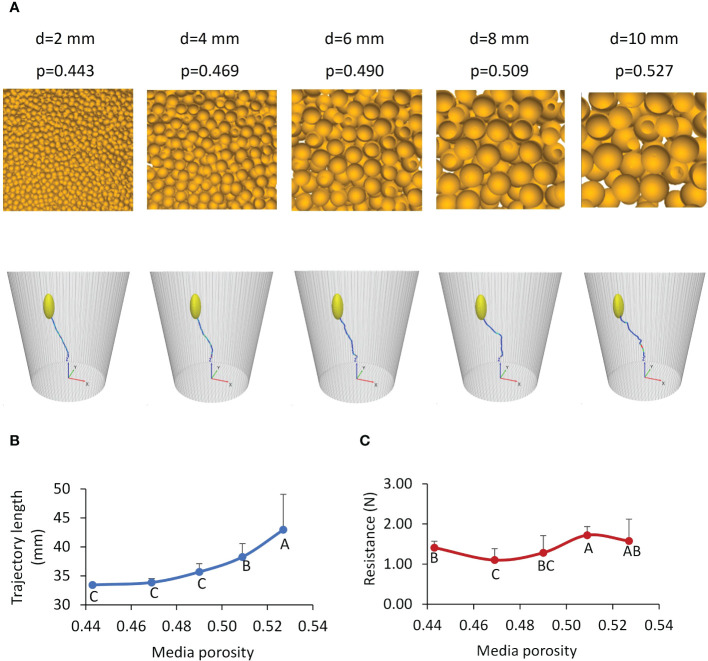
Effects of growth medium conditions. **(A)** Simulated growth media with various porosities (p) created using different particle diameters (d), and the corresponding trajectories of cotyledon. **(B, C)** Simulated total length of trajectories and average soil resistances to the cotyledon, respectively; values labeled with different letters are statistically different according to Duncan’s Multiple Range Test at P<0.05; the error bars stand for standard deviations.

Increased particle size resulted in increased porosity, as visually seen in **Figure 12A**. The trajectory of the path was wavier in the growth medium with a higher porosity (**Figure 12A**). Since medium particles were randomly packed, the distributions of pores in the medium were also random. Therefore, the variation of the path did not have any particular pattern. However, the total trajectory path length showed a defined, non-linear increasing trend with the medium porosity (**Figure 12B**). The passing-route that connects large pores must be longer; thus, the cotyledon travelled a greater distance along the route to the surface. In terms of resistance, pushing larger particles takes a greater force, but the cotyledon may not need to push the large particles if pores allowed for an alternative path in the high-porosity medium. The contradicting factors of large particles (hard to push particles) and large and increasing numbers of pores confound each other, resulting in no particular patterns of resistances (**Figure 12C**).

#### 3.2.4 Dynamics of seedling in Lunar soil

**Figure 13:**
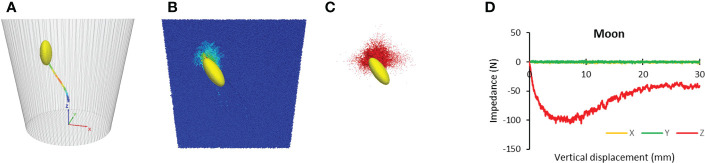
Simulated paths and resistances for Lunar soil. **(A)** Trajectory. **(B)** Particle velocity contours of soil particles. **(C)** contact force chains within the soil particles. **(D)** Resistances to cotyledon.

Results showed that for Lunar soil, cotyledon also followed a natural none-straight path (**Figure 13A**), suggesting that the seedling took the path of least resistance. The cotyledon impacted the overlaying soil particles, as shown by the particle velocity contour (**Figure 13B**), while advancing toward the soil surface. As a result, contact forces arose among those particles, forming a network of force chains (**Figure 13C**). These phenomena are similar to those observed in terrestrial soil. However, the impact zone was smaller in the Lunar soil than that in terrestrial soil shown in **Figures 11A, B**. The force-displacement curve of Lunar soil was smoother than that of terrestrial soil (**Figure 13D**), possibly due to the smaller particle size of the Lunar soil. The trajectory length of cotyledon in the Lunar soil was 35.3 mm which was longer than that of terrestrial soil at 33.6 mm. The most important information was that the average impedance of Lunar soil was 64.2 N which was extremely high.

### 3.3 Limits of plant life

**Figure 14:**
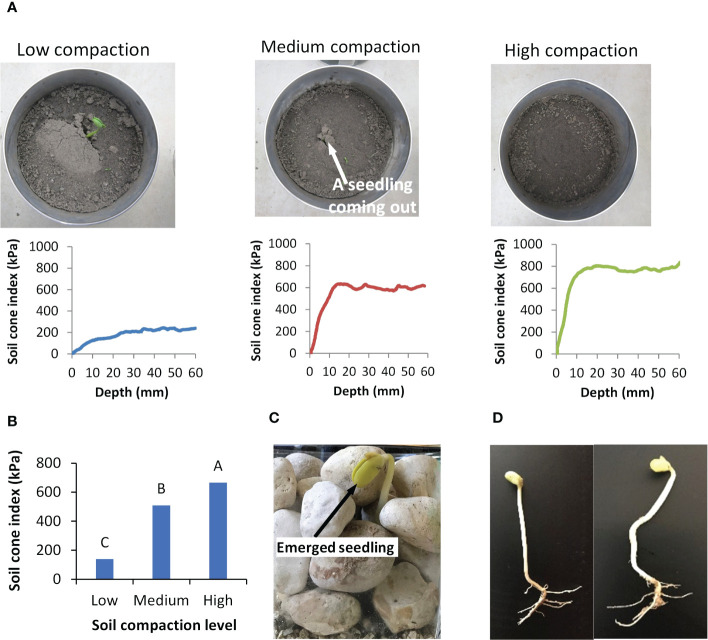
Effects of growth medium on seedling emergence. **(A)** Seedling emergence in soils with three different compaction levels and the corresponding soil cone indices measured using the micro-penetrometer. **(B)** Comparisons in average soil cone indices under different soil compactions; values labeled with different letters are statistically different according to Duncan’s Multiple Range Test at P<0.05; error bars stand for standard deviations. **(C)** Photo of a soybean seedling emerged from gravel. **(D)** A straight and a bent soybean seedling emerged from gravel.

Seedlings first emerged from the Low compacted soil (**Figure 14A**), followed by the emergence in the Medium compaction soil, and finally, no emergence from the High compacted soil. Measurements of soil resistance using the micro-penetrometer explains these results (**Figure 14A**). The soil cone indices increased from the surface to a higher level in the more compacted soil. In other words, compacted soil had decreased penetrability. The differences in soil cone index between the High compaction soil and the other two compaction soils were significant (**Figure 14B**). In examination of role of soil porosity, the highly compacted soil had reduced porosity. without the availability of large pores in any direction, the seedling would need to grow straight upward. In this case, the penetrability of the medium governs the emergence. When the overlaying compacted soil was impenetrable, the seedling was not able to emerge.

However, we observed that 17% of the seedlings were able to emerge from the gravel medium (**Figure 14C**). If a seedling grew straight up, the gravel medium would be impenetrable. Since the large gravel pieces functioned as physical barriers, the emerged seedling would not be able to push the overlaying particles. In nature, the seedling waved through the heavy particles. These emerged seedlings must have travelled through a series of connected pores that formed a continuous channel. Because of the irregular shapes of gravel pieces, some channels might be straighter and shorter, while some others might be more curved and longer. This helps clarify why some seedlings turned out straight, while others, bent (**Figure 14D**). Seedlings that did not make it to the surface are presumed to have died, either because their path through the channels ended abruptly or because the seedling’s energy reserve has been exhausted from the lengthy journey. It is commonly known that a “hard” growth medium may prevent the seedling from emerging (Rathore et al., 1981; Hyatt et al., 2007). Both the compacted soil and gravel are considered to represent such growth medium. However, the soybean seedling responded to each medium differently.

## 4 Discussion

A dark-grown seedling must acquire abilities to emerge from underground in order to establish life on land. This involves the ability to pursue the path of least resistance while manipulating its morphological characteristics in space and time, highlighting some of the many important survival strategies adopted in seed-producing plants. The dark-grown seedling shaped and orientated itself to gain mechanical advantages. The cotyledon, a large embryonic appendage, does not lead the way through the growth medium. Instead, the hypocotyl, shaped as a “wedge”, leads the way and pull the cotyledon upward to the surface (**Figure 6A**). Mechanically, a wedge shape (angled hypocotyl) is advantageous over a blunted shape (cotyledon) in penetrating a medium. As the seedling grows, the cotyledon rotates itself to obtain a position that has a smaller pressure area (**Figure 6C**). This not only reduces the resistance to the seedling, but also reduces the risk of self-damage to the cotyledon, a crucial source for nutrients to sustain the growth of dark-grown seedling (Harris et al., 1986; Ishida et al., 1990).

The path taken by seedlings follows an overall upward direction, with the guidance of gravitropism. However, the seedling circumnavigates the obstacles within the non-homogenous growth medium (**Figures 8**, **10**) in pursuing the path of least resistance. Therefore, the path of least resistance is not necessarily the shortest path, depending on the condition of growth medium. Thus, there is a trade-off between pushing to overcome the overlying resistance or to travel a longer distance. To date, it has yet to be elucidated how a seedling makes this decision in selecting a path. In studying the biological effects of a seedling’s intrinsic signals on growth direction, Gupta et al. (2012) reports that the optimal hypocotyl growth direction should be the easiest and shortest route. However, our findings suggest that a seedling does not select the optimal route. Instead, it chooses the path of least resistance, regardless its length (**Figure 12**). Initially, the seedling may attempt to grow straight up, governed by gravitropism. When the seedling subsequently comes into contact with overlaying medium particles, it turns toward a direction with less resistance. This process repeats itself until either the seedling finally surfaces, or until its energy is depleted.

A small seedling is sometimes able to emerge from a pile of rocks, while in other times it cannot even break through a mere mound of soil. The results of this study help elucidate this perplexing phenomenon. In the former case, pore availability between rocks is critical to allow the seedling to pass through the channels formed by the pores (**Figure 14C**). In the latter case, the highly resistant soil holds its particles tightly by cohesion and friction, becoming mechanically impenetrable to the seedling in the vertical direction, and lacking pores in lateral directions. In such a case, seedlings are neither able to find pores to pass, nor able to push the overlaying soil particles, thus failing to emerge (**Figure 14A**). This suggests that pore availability and penetrability of growth medium or the combination of both limiting factors to plant life.

The seedling’s destiny is ultimately in the hands of the growth medium. The results of our study demonstrate that, on terrestrial soil, pore availability and penetrability of growth medium are two limiting factors in seedling emergence. This fundamental concept can be applied on both earth and in space. Using simulants as growth medium, researchers have found that plants could indeed grow in space (Wamelink et al., 2014; Wamelink et al., 2019). However, extra-terrestrial soil is fine in particle size and heavy in particle density (Wood, 1970; Hender, 2007). The simulation of Lunar soil showed that cotyledon travelled a 5% longer trajectory than in the terrestrial soil. However, the soil impedance of Lunar soil was 46.5 times of the terrestrial soil. This suggests that a seedling may not be able to overcome such a high soil impedance on the Moon. Therefore, to grow crops on the Moon, conditioning soil, for example, increasing soil porosity, may be a necessary step prior to planting any seeds. A dark-grown seedling has the ability to adjust its growth direction and optimize its morphology, which are both impressive adaptations for successful emergence. Future research is warranted to experimentally examine the feasibility of seedling emergence in extra-terrestrial soil.

## 5 Conclusions

Using soybean as a case study, we first took CT scans of dark-grown seedlings in terrestrial soil to examine the growth direction and the morphological changes of the seedling during emergence. Then, we carried out numerical modelling to simulate the growth of a dark-grown seedling in a growth medium. Finally, we examined the limiting factors to plant life, namely soil pores and penetrability. We found that the seedling shapes and orients itself to obtain mechanical advantages for overcoming resistance in the growth medium while maintaining self-protection. We also found that a dark-grown seedling took a weaved path on its journey to the earth surface. We demonstrated that the path of least resistance, a fundamental law of nature, applies to a dark-grown seedling in searching for the path. Selecting the path of least resistance may be at the expense of traveling longer distances. Both pore availability and penetrability of the growth medium play a role in determining the fate of a dark-grown seedling. A growth medium with low penetrability, lacking large pores, or a combination of these, may prematurely halt the seedling’s journey to emergence. The finer particles of Lunar soil means less pores, and the heavier particle density of Lunar soil means a lower penetrability. This makes Lunar soil be less favorable for seedling emergence. Future research is warranted to experimentally examine the feasibility of seedling emergence in extra-terrestrial soil. Our results suggest that the path of least resistance and the mechanically advantageous morphology may have had evolutionarily adaptive benefits to the survival of seed-producing plants on land. This understanding of this has implications in future efforts of growing seed-plants in alternative soils, including extra-terrestrial soil.

## Data availability statement

The original contributions presented in the study are included in the article/supplementary material. Further inquiries can be directed to the corresponding authors.

## Author contributions

HG performed the literature review and data analysis, as well as compiled the results. LQ and YC oversaw the work and contributed to methodology development and result interpretation. ZZ, HG, and LG developed the model and run the simulations. LT contributed to manuscript writing, editing, and result and discussion. SL and ZY performed image analysis and data processing. ZZ and WZ conducted CT scans. All authors contributed to the article and approved the submitted version.
